# The impact of health literacy on shared decision making before elective surgery: a propensity matched case control analysis

**DOI:** 10.1186/s12913-018-3755-9

**Published:** 2018-12-12

**Authors:** Gildasio S. De Oliveira, Martin Errea, Jane Bialek, Mark C. Kendall, Robert J. McCarthy

**Affiliations:** 1 Department of Anethesiology, Alpert School Of Medicine, Brown University, 593 Eddy Street, Davol 129, Providence, Rhode Island USA; 2 Department of Health Services Research, Practice and Policy, School of Public Health, Brown University, Providence, Rhode Island USA; 3 Department of Radiology, Northwestern University, Feinberg School of Medicine, Chicago, Illinois USA; 4 University of Illinois at Chicago, School of Medicine, Chicago, Illinois USA; 5 Department of Anesthesiolgy, Rush University Medical Center, Chicago, Illinois USA; 6 Department of Surgery, Alpert School of Medicine, Brown University, Providence, Rhode Island USA

**Keywords:** Health literacy decision conflict elective surgery

## Abstract

**Background:**

Poor health literacy affects over 90 million Americans. The primary aim of the study was to evaluate a possible association between health literacy and decision conflict in surgical patients.

**Methods:**

Patients undergoing a diverse number of elective surgeries were enrolled in the study. Health literacy was measured using the Newest Vital Sign instrument and decision conflict using the low literacy version of the Decision Conflict Scale.

**Results:**

200 patients undergoing elective surgeries were included in the study. Patients who had greater health literacy scores had lower decision conflict scores, Spearman’s rho = − 0.43, *P* < 0.001. Following propensity-score matching to account for potential covariates, the median (IQR) decision conflict score was 20 (0 to 40) for patients with poor health literacy compared to 0 (0 to 5) for patients with adequate literacy, *P* < 0.001.

**Conclusions:**

Poor health literacy is associated with greater decision conflict in patients undergoing elective surgical procedures. Strategies should be implemented to minimize decision conflict in poor health literacy patients undergoing elective surgical procedures.

## Background

Health literacy is defined as the ability to obtain, read, understand, and use healthcare information in order to make appropriate health decisions and follow instructions for treatment [1]. More than 90 million Americans have poor health literacy [1]. Poor health literacy has been associated with poor health outcomes (e.g., hospitalizations, readmissions and mortality) in medical patients [2–4]. Health literacy directly affects patients’ ability to navigate complex health care systems, to make informed decisions and provide self-care [5]. It is, therefore, expected that poor health literacy can have a large impact on the care and health outcomes of surgical patients who often need to follow important instructions during the perioperative phases [6–9]. Nonetheless, our group has demonstrated that studies evaluating the impact of poor health literacy on the care of surgical patients are vastly scarce [10].

The decision making process for surgical patients undergoing various elective surgeries for different conditions frequently involves uncertainties that can lead to decisional conflict [11]. The simple lack of comprehension by patients regarding surgical risks and benefits is a major contributor to the development of decision conflict and poor shared decision making [12–14]. It has been demonstrated that poor health literacy influences decision making in non-surgical settings, but it is unknown the extent to which patients with poor health literacy are at risk for greater decision conflict before surgery.

The primary purpose of the study is to explore the association between health literacy and decision conflict in surgical patients undergoing a diverse number of elective surgeries. We hypothesized that patient with poor health literacy would have greater decision conflict than patients with adequate health literacy. In addition, we also sought to explore a potential accord between the patients’ overestimation of surgical mortality risk and the decision conflict prior to elective surgeries.

## Methods

This cross-sectional study included consecutive English speaking patients eighteen years or older who were scheduled to undergo elective surgeries. This study received institutional review board approval by Northwestern University. The work adheres in line with the STROCSS criteria and was registered at http://www.researchregistry.com, unique identifier number: researchregistry3296 [15]. Each participant provided written informed consent prior to enrollment. Patients were excluded if they had ailments that could affect communication (e.g., uncorrected visual and/or hearing impairment, aphasia), Alzheimer’s disease and if they were undergoing oncological or emergency procedures.

Patients undergoing elective surgical procedures were evaluated in the preoperative characteristics (e.g., age, gender, race), 2) American Society of Anesthesiology (ASA) physical classification, 3) medical history, 4) educational level, 5) annual income, and 6) surgical procedure specialty.

The questionnaires were administered to patients in the preoperative holding area face to face by a research assistant. Patients’ health literacy was evaluated using a specific, validated tool, called the Newest Vital Sign (NVS) [16]. The NVS is a 6-item assessment of health literacy and numeracy using questions about an ice cream nutrition label. It is a reliable (Cronbach’s alpha in 3 studies range from 0.74–0.81) screening tool used to determine risk for poor health literacy. It includes computational skills as well as reading comprehension, and is felt to assess more complex cognitive functions than shorter word-recognition tests of literacy. Patients are given an ice cream nutrition label and are asked 6 questions about how they would interpret and act on the information contained on the label. Each question correctly answered is given one point; Scores ≤3 indicates poor health literacy, while scores ≥4 indicates adequate health literacy. The NVS has been shown to have a high sensitivity for predicting poor health literacy skills and has a strong correlation with other established measures, such as the REALM and TOFHLA [17].

Decision conflict was evaluated using the low literacy version of the decision conflict scale which is a validated tool that consists of 10 questions [18]. Each question is scored using yes = 0, unsure = 2 and no = 4. The values are then summed and multiplied by a factor of 2.5. Scores from the scale range from Zero (no decisional conflict) to 100 (extremely high decisional conflict). A total score greater than 30 has been considered high decisional conflict in healthcare as this score may imply that patients were unsure in more than 5 out 10 questions of the questionnaire [19, 20].

Patients were asked to give an estimate on the chance of dying as a result of the surgical procedure. The probability of postsurgical mortality was determined by using the web-based online American College of Surgeons NSQIP surgical risk calculator [21]. The surgical risk calculator is an on-line tool created in 2013 to improve communication between surgeons and patients by proving a customized risk assessment of undesirable surgical postoperative outcomes including mortality. The calculator has outstanding properties when assessing mortality (c-statistic = 0.944; Brier score = 0.011 [where scores approaching 0 are better]). Overestimation of mortality probability was obtained through the difference between the patient’s estimation of surgical mortality and the value obtained by American College of Surgeons NSQIP surgical risk calculator.

We performed a power analysis that estimated a sample size of 193 patients would be required to achieve 80% power to detect a difference of − 0.2 between the null hypothesis correlation of 0.0 and the alternative hypothesis correlation of 0.2 for the association between health literacy and decision conflict using a two-sided hypothesis test with a significance level of 0.05. Continuous data were analyzed using independent t-tests or Mann-Whitney U test as appropriate. Categorical data were analyzed using the Fisher’s exact test.

Spearman’s rank correlation coefficient was used to measure an association between mortality risk overestimation and decision conflict. A propensity score matching analysis was conducted to confirm the relationship between health literacy and decision conflict. Variables that were associated with poor shared decision making in other clinical settings or that were linked with decision conflicts in the current dataset (*P* < 0.2) were included in the analysis.

The propensity score is the conditional probability for patients who experienced high decision conflict (≥30) and for those who did not have a high decision conflict (< 30) as a function of predefined covariates which was added into a multiple logistic regression. Continuous variables were dichotomized by analyzing their discriminant properties following the construction of receiver operating curves for decision conflict. Individual propensity scores derived from the logistic regression analysis were calculated.

The propensity scores were used to create a one to one matched analysis (nearest neighbor with caliber matching) of patients who had poor health literacy with patients who did not have poor health literacy by a random selection. Following the Cochran and Rubin algorithm, we used a caliper width of the pair to be within 0.6 standard deviations (SD) to match the pairs [22]. Patients who did not follow within the caliper width was excluded. Simple bivariate analysis was used with the matched groups to examine an independent association among patients with poor health literacy and high decision conflict. A two-sided *p* value < 0.05 was considered statistically significant.

## Results

Among 218 patients undergoing elective surgical procedures who were screened between June 2014 and September 2014, 200 met our inclusion criteria. Patients were undergoing large and diverse types of elective surgeries (Table 1). 63 out of 200 (32%) patients had poor health literacy. Poor health literacy was more common in older, non-White, poorly educated and lower income patients (Table 2).

**Table 1:** Number of Cases by Surgical Specialties

Specialty	Total (*n* = 200)
Orthopedics	50 (25%)
General Surgery	43 (21.5%)
Ear Nose and Throat	26 (13%)
Urology	26 (13%)
Gynecology	14 (7%)
Vascular	7 (3.5%)
Spine	14 (7%)
Plastics	16 (8%)
Ophthalmology	4 (2%)

**Table 2:** Demographic factors between patients with poor and adequate health literacy

	Poor HL (*n* = 63)	Adequate HL (*n* = 137)	*P* value
Age (years)	58 ± 16	49 ± 15	0.0004
Gender			
Male	31(49%)	79(58%)	0.29
Female	32 (51%)	58 (42%)	
Race			
Caucasian	32 (51%)	105 (77%)	0.001
Non- Caucasian	31(49%)	32 (23%)	
Education			
High school or less	28 (44%)	16 (12%)	< 0.001
College/Graduate	35 (56%)	121(88%)	
Yearly Income			
≤50,000	20(32%)	19 (14%)	0.004
> 50,000	43(68%)	118 (86%)	
ASA Class			
I/II	48(76%)	118(86%)	0.11
III/IV	15(24%)	19(14%)	

*ASA* American Society of Anesthesiology Physical Classification

*HL* Health Literacy

**Fig. 1:**
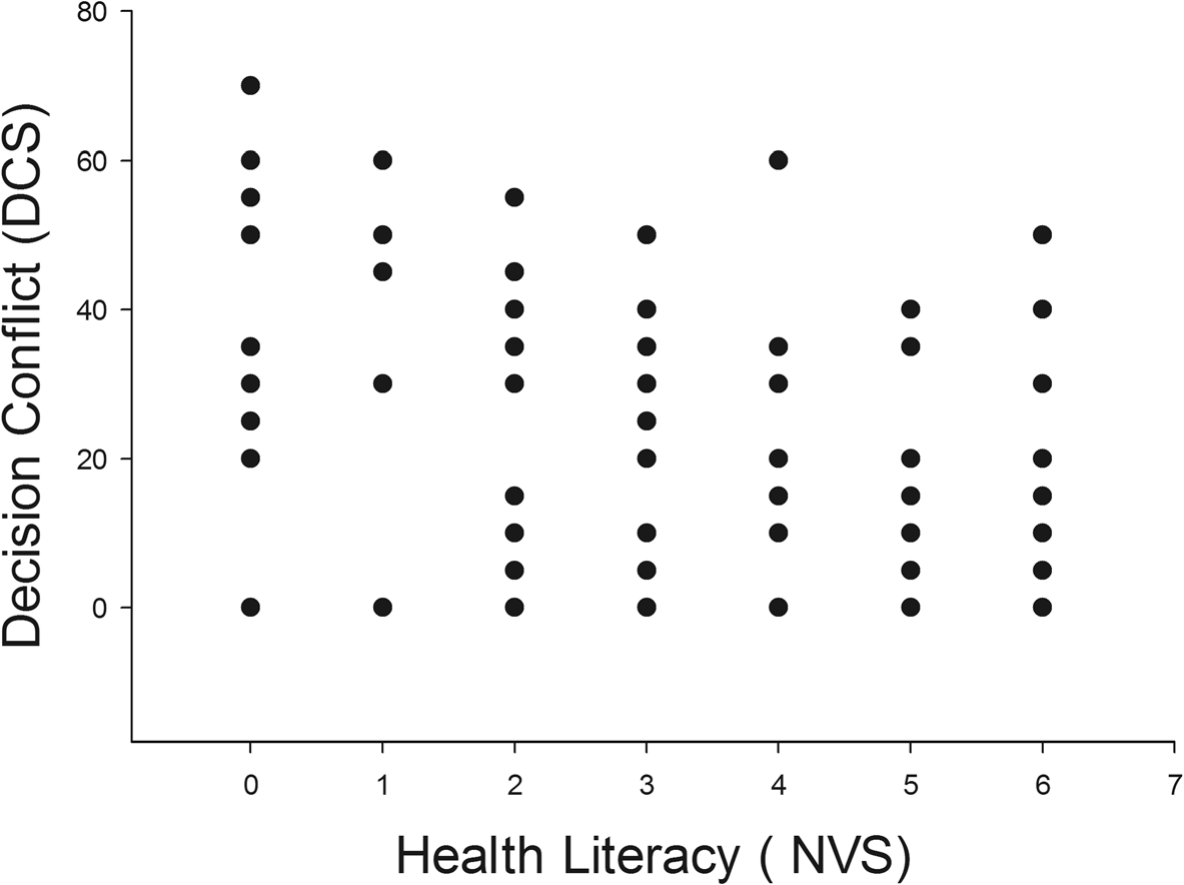
Scatter plot demonstrating an inverse relationship between health literacy and decision conflict scores. Spearman’s rho = − 0.43, *P* < 0.001

There was an inverse correlation between health literacy scores and decision conflict score. Patients who had greater health literacy scores had lower decision conflict scores (Fig. 1, *P* < 0.001). The median (IQR) for decision conflict score in patients with poor health literacy was 30 (0 to 45) compared to 0 (0 to 5) in patients with adequate literacy, *P* < 0.001.

Forty-seven patients with poor health literacy were propensity matched to 47 patients who had adequate health literacy. Confounding covariates were similarly distributed in the propensity matched groups (Table 3). In the propensity matched groups, the median (IQR) decision conflict score was 20 (0 to 40) for patients with poor health literacy compared to 0 (0 to 5) for patients with adequate literacy, *P* < 0.001. Twenty five out of 47 (53%) patients with poor health literacy had high decision conflict compared to 5 out 47 (11%) patients with adequate literacy, *P* < 0.001.

**Table 3:** Covariate distribution between propensity matched groups

	Poor HL (*n* = 47)	Adequate HL (n = 47)	*P* value
Propensity Score	0.392 ± 0.20	0.392 ± 0.20	0.99
Age (years)			
< 54	19 (40%)	20 (43%)	1.0
≥54	28 (60%)	27(57%)	
Gender			
Male	22(47%)	23 (49%)	1.0
Female	25(53%)	24 (51%)	
Race			
Caucasian	20 (43%)	20 (43%)	1.0
Non- Caucasian	27 (57%)	27(57%)	
Education			
High school or less	15(32%)	16 (34%)	1.0
College/Graduate	32(68%)	31 (66%)	
Yearly Income			
≤50,000	11(23%)	11(23%)	1.0
> 50,000	36(77%)	36 (77%)	
ASA Class			
I/II	35 (74%)	38 (81%)	0.62
III/IV	12 (26%)	9 (19%)	

*ASA* American Society of Anesthesiology Physical Classification

*HL* Health Literacy

There was not a significant correlation between overestimation of surgical risk and the decision conflict, rho = 0.06, *P* = 0.47. Eleven from 29 subjects who overestimated mortality risk by ≥5% had high decision conflict compared to 30 out 119 who overestimated surgical risk < 5%, *P* = 0.17.

## Discussion

The most important finding of the current study is the robust association between health literacy and decision conflict in patients undergoing elective surgical procedures. Patients with poor health literacy had greater decision conflict when compared to patients with adequate health literacy. The association was still detected even after it was adjusted for potential confounding covariates (e.g., education, race, income) using propensity matching analysis. Taken together, our results suggest that patients with poor health literacy are likely at risk for poor shared decision making before elective surgeries.

Our results are clinically important since shared decision making is considered an important strategy to improve quality of care, while still reducing healthcare costs [23–25]. Furthermore, shared decision making has been considered the pinnacle of patient-centered care [26, 27]. Since it is estimated that over 90 million Americans have poor health literacy, targeted efforts to improve shared decision making for patients with poor health literacy undergoing elective surgery can improve quality of surgical care in the United States. To the best of our knowledge, this is the first study to detect health literacy as an important factor for shared decision making in surgery.

Another important finding was the lack of correlation between patients’ estimation of surgical risk and decision conflict. The creation of precise risk estimation (such as the NSQIP risk calculator used in this study) represents an important effort to promote shared decision making in surgical patients [28–32]. Nonetheless, our current results suggest that precise risk estimation alone may not always lead to shared decision making. Patients with poor health literacy may not comprehend surgical risk estimates as it is currently presented in the clinical settings. Improvements in risk communication strategies targeting the needs of patients with poor health literacy before surgery are warranted.

The use of decision aids results in a small (− 5.0, 95%CI: -7.1 to − 2.9 points), but effective strategy to reduce decision conflict in patients facing a surgical treatment [33]. Nevertheless, the effect of decision aids on decision conflict is not entirely clear for patients undergoing elective surgical procedures as four studies showed benefit and two did not in a recent systematic review [34]. Another systematic review demonstrated that most studies do not incorporate health literacy best practices when developing decision aids for patients [35]. Our current results support the need to develop decision aids using health literacy best practices for patients undergoing elective surgeries.

Recently, the American College of Surgeons (ACS) and National Institutes of Health (NIH) convened a research summit to develop a national surgical disparities agenda [36]. The improvement of patient clinical communication was described as a major priority, specifically the need to address patients’ health literacy and decision making. The current investigation reinforces the need to improve patient-clinician communication and the potential role of health literacy in mediating disparities in surgical care.

Although we studied primarily the impact of health literacy on share decision making during elective surgery, it is possible that health literacy may have an important impact on other phases of the surgery. Patients who do not understand preoperative instructions may not follow them and this may result in cancellations [37, 38]. Patients who do not understand how to take postoperative pain analgesics may develop greater postoperative pain than patients who take their medications appropriately after surgery [39, 40].

Our study suggests the need to improve preoperative communication between health care providers (e.g., surgeons, nurses) and patients. This could be achieved by incorporating decision aids into the preoperative workflow, preferably during the pre-anesthesia evaluation many days before the day of surgery. This would allow patients to consider their options, ask questions, discuss the process and follow up care over time.

This investigation must be interpreted according to its limitations. We only studied patients undergoing non-cancerous elective surgical procedures, and we cannot generalize our findings to patients undergoing oncologic or emergent surgery. Nonetheless, we hypothesize that due to time constraints, patient-clinician communication in oncologic and emergent surgery can be severely jeopardized with greater implications to shared decision making. Although a positive correlation between decision conflict and regret has been established in other clinical scenarios, we did not evaluate long term decision regret in the current study population [28]. Future studies assessing shared decision making in surgical patients would benefit from including a longitudinal evaluation of patients’ decision regret.

## Conclusions

Shared decision making is considered a key component of patient-centered care. We demonstrate that patients with poor health literacy are more likely to have decision conflict before elective surgeries. Targeting patients with poor health literacy (approximately 90 million American) can be an important strategy to improve shared decision making and, ultimately, the overall quality of care experienced by surgical patients.

## Abbreviations

ACS: American college of surgeons
ASA: American Society of Anesthesiology
CI: Confidence interval
DCS: Decision Conflict Scale
IQR: Interquartile range
NVS: Newest Vital Sign
REALM: Rapid Estimate of Adult Literacy in Medicine
SD: Standard deviation
STROCSS: Strengthening the Reporting of Cohort Studies in Surgery
TOFHLA: Test of Functional Health Literacy in Adults
